# Role of Mechanical Circulatory Support in Complex High-Risk and Indicated Percutaneous Coronary Intervention: Current Indications, Device Options, and Potential Complications

**DOI:** 10.3390/jcm13164931

**Published:** 2024-08-21

**Authors:** Francesca Maria Di Muro, Michele Bellino, Luca Esposito, Tiziana Attisano, Francesco Meucci, Alessio Mattesini, Gennaro Galasso, Carmine Vecchione, Carlo Di Mario

**Affiliations:** 1 Department of Experimental and Clinical Medicine, School of Human Health Sciences, Careggi University Hospital, University of Florence, 50134 Florence, Italy; 2 Department of Medicine, Surgery and Dentistry, University of Salerno, 84084 Baronissi, Italy; mbellino@unisa.it (M.B.); tiziana.attisano@gmail.com (T.A.); ggalasso@unisa.it (G.G.); cvecchione@unisa.it (C.V.); 3 Department of Advanced Biomedical Sciences, University Federico II, 80138 Naples, Italy; lucaesposito1293@gmail.com; 4 Division of Structural Interventional Cardiology, Cardiothoracovascular Department, Careggi University Hospital, 50134 Florence, Italy; francescomeu19@gmail.com (F.M.); amattesini@gmail.com (A.M.); carlo.dimario@unifi.it (C.D.M.); 5 Vascular Pathophysiology Unit, IRCCS Neuromed, 86077 Pozzilli, Italy

**Keywords:** mechanical circulatory support, CHIP, PCI

## Abstract

Improved expertise and technological advancements have enabled the safe and effective performance of complex and high-risk-indicated percutaneous coronary intervention (CHIP) in patients previously considered inoperable or high-risk. Mechanical circulatory support (MCS) devices play a crucial role in stabilizing hemodynamics during percutaneous coronary intervention (PCI) -related ischemia, thereby reducing the risk of major adverse events and achieving a more complete revascularization. However, the use of MCS devices in protected PCI is not without risks, including peri-procedural myocardial infarction (MI), bleeding, and access-related complications. Despite numerous observational studies, there is a significant lack of randomized clinical trials comparing different MCS devices in various CHIP scenarios and evaluating their long-term safety and efficacy profiles. This review aims to summarize the current evidence regarding the benefits of MCS devices during CHIPs, offer a practical guide for selecting appropriate devices based on clinical scenarios, and highlight the unanswered questions that future trials need to address.

## 1. Introduction

Over the past few decades, advancements in technology and increased expertise among interventional cardiologists have significantly expanded the indications for PCI, enabling the performance of increasingly complex and high-risk procedures. This includes those patients with an indication of Coronary artery bypass graft surgery (CABG) that have been deemed inoperable or high surgical risk due to procedural or clinical factors such as unsuitable CABG targets, a porcelain aorta, or significant comorbidities [1,2].

**Figure 1:**
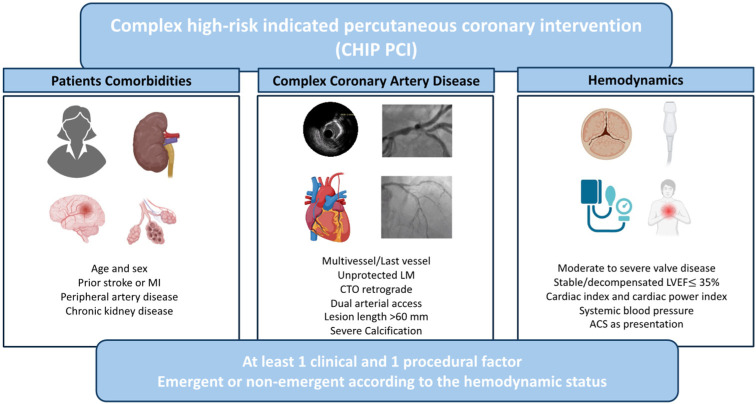
Complex high-risk indicated percutaneous coronary interventions according to patient clinical characteristics and comorbidities, complexity of coronary artery disease and hemodynamics.

To match the CHIP definition, at least one anatomic and one clinical factor are required, thereby including different patient profiles ranging from those with unstable presentations, such as cardiogenic shock or acute coronary syndromes, to more stable scenarios (Figure 1) [3,4,5]. Therefore, a multidisciplinary heart team is crucial for categorizing procedures as CHIPs and collegially deciding the best treatment strategy. When the indication is placed, these procedures often require a longer fluoroscopy time, a higher amount of contrast, and extensive lesion preparation using prolonged high-pressure balloon inflation or direct calcium ablation techniques with more aggressive devices, like orbital or rotational atherectomy. In such cases, MCS may be useful in ensuring hemodynamic stability throughout the procedure and reducing the risk of complications. In this review, we aim to provide an overview of the most commonly used MCS devices in CHIPs, describing typical scenarios as well as frequent complications. Additionally, we discuss a simple and useful decision-making algorithm for the interventional cardiologist while highlighting the potential unmet needs that current and future research aim to address.

## 2. Available MCS Devices and Their Rationale

### 2.1. Type of MCS Devices

**Figure 2:**
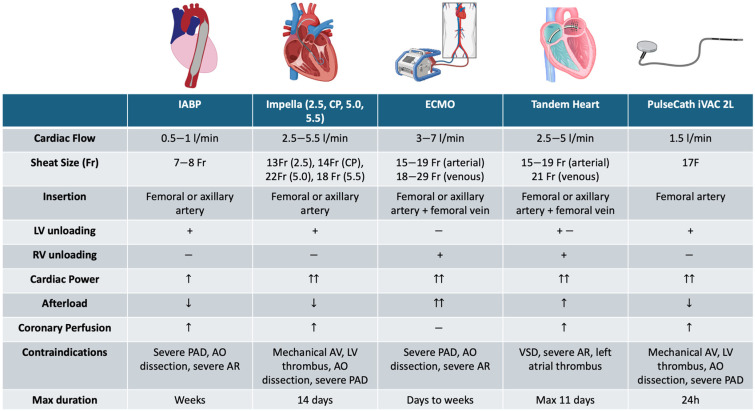
Mechanical Circulatory Support Devices for Complex High-risk Coronary Intervention.

MCS devices can be classified according to their mechanism of function and duration of use. The main characteristics of those in current clinical use are summarized in Figure 2 and Table 1.

The intra-aortic balloon pump (IABP) has an indirect mechanism of action, inflating during diastole, synchronously with aortic valve closure, and deflating rapidly just before systole. This way, it reduces afterload and allows blood displacement from the aorta to the peripheral vascular district, resulting in a modest increase in cardiac output (0.5 L/min) and improved coronary perfusion through diastolic aortic pressure augmentation. It is inserted via femoral access and is available in different sizes according to body size area.

The IABP is currently the most widely used MCS device due to the extensive experience gained, ease of placement and management, and low complication rates [6,7]. However, the only adequately powered randomized trial (RCT), the Balloon Pump Assisted Coronary Intervention Study (BCIS-1), which compared IABP-assisted PCIs with control patients, failed to show any reduction in myocardial infarction or mortality at 28 days despite more prolonged hypotension (10.7% vs. 1.3%, *p* < 0.001) and higher rates of rescue IABP placement (up to 12%) in the control arm [8]. Therefore, according to current European guidelines, an IABP should not be employed in CHIP procedures [9].

Microaxial Flow Pump (Impella): This axial device leverages the principles of Archimedes’ screw and transfers blood from the left ventricle to the ascending aorta with a support capacity ranging from 2.5 to 5.5 L per min [10]. Both Impella 2.5 and Impella CP are designed to be placed percutaneously through femoral or axillary access with peel-away sheaths, while Impella 5.0 and 5.5 require surgical exposure of the femoral or subclavian/axillary artery. The device is advanced into the ascending aorta and across the aortic valve deployed into the left ventricle [11,12]. Its primary hemodynamic effects include direct LV unloading achieved by decreasing left ventricular (LV) end-diastolic pressure, LV workload, and myocardial oxygen demand, as well as improving coronary artery perfusion and cardiac output. However, major limitations include the lack of active oxygenation and the need for adequate pre-load to ensure LV filling, which can be compromised by hypovolemia or right ventricular failure. In such situations, after addressing the underlying issues, Extracorporeal Membrane Oxygenation (ECMO) or a combination of ECMO and Impella (known as ECPELLA) should be considered to provide simultaneous systemic circulation support and LV unloading [13,14].

The efficacy of Impella in CHIPs has been demonstrated in several observational registries and in the large PROTECT II RCT. This study, comparing IABP and Impella 2.5 in 452 non-emergent high-risk patients, showed similar outcomes at 30 days while showing fewer major adverse events (MAEs) in the Impella group at 90 days (*p* = 0.023) due to less hypotensive events, higher cardiac power output, and facilitation of more extensive atherectomies. Interestingly, the difference in long-term outcomes disappeared in the per-protocol analysis population of 427 patients [15]. A first explanation to these results may be a bias in the selection of the patient population, determining an attenuation of the beneficial effects of the Impella. Indeed, when limiting the population to 325 very high-risk patients with multivessel disease and severe LV dysfunction in a PROTECT II sub-study, the MAE rates were significantly lower in the Impella 2.5 group compared to IABP at 90 days [16]. Another reason might be the lower efficacy of older-generation devices employed in this trial. A retrospective analysis of 198 elective high-risk PCI patients (35% using new-generation MCS and 65% without support) demonstrated a better safety and efficacy profile for new-generation devices (PulseCath iVAC2L, Impella CP, and HeartMate PHP) [17]. Similar results were achieved in the PROTECT III trial, which tested a new-generation device, Impella CP, in 318 PROTECT II-like patients with severely depressed LVEF undergoing PCI under Impella CP support versus a matched cohort of 216 PROTECT II patients. In-hospital outcomes were in favor of Impella CP with lower rates of MAEs, MI, bleeding, hypotensive episodes, and arrhythmias [18].

The ongoing PROTECT IV (NCT04763200) trial aims to provide further insight into MCS’s employment answering the still pending questions on MCS in patients with complex CAD and impaired LV function undergoing a protected PCI with Impella support vs. standard of care with or without an IABP.

ECMO is typically selected in cases of respiratory failure with or without extremely severe cardiac shock. However, it has also been described as a feasible strategy for achieving complete revascularization in CHIPs. There are two types of ECMO, namely Veno-venous ECMO or Veno-Arterial ECMO. Veno-venous ECMO extracts blood from the vena cava or right atrium and returns it to the right atrium. Extraction and reinfusion can occur by single or double cannula. It only provides respiratory support and requires stable hemodynamics to be used. Conversely, Veno-Arterial ECMO performs drainage from the femoral vein and perfusion through the femoral, axillary, or carotid artery and provides complete cardiac and respiratory support. Complications of ECMO are common and, as reliable, strongly associated with an increase in morbidity and mortality [19,20]. Evidence on VA-ECMO-supported CHIPs is limited to small retrospective studies, which show nearly a 17–18% reduction in major adverse cerebrovascular and cardiovascular events and 0–7% mortality rates in the VA-ECMO group compared to the non-protected one [21,22]. However, since no RCT or meta-analysis data are available, current guidelines can only be based on expert consensus and do not recommend its use in CHIPs.

TandemHeart is performed through a transseptal inflow cannula that aspirates blood from the left atrium, subsequently pumping into the femoral artery. The whole system is driven by an electromagnetic motor which provides a flow ranging from 3.5 L/min to 4.0 L/min at a maximum speed of 7500 rpm. Such a system involves a transseptal puncture that requires an experienced operator and trained staff to be safe and effective. Generally, the highest level of guaranteed support results in better hemodynamics, peripheral perfusion, and metabolic control, even though clear evidence of better outcomes has not yet been recognized in RCTs [23].

Finally, among the new generation of LV assist devices, the PulseCath iVAC 2L stands out. It is a membrane pump connected to a bi-directional flow catheter and driven by an IABP console, and it generates a blood flow of up to 1.5 L/min. It is inserted through the femoral artery through an 18 F sheet and advanced retrogradely across the aortic valve into the left ventricle. It increases the mean systemic arterial pressure and cardiac output while not influencing the mean pulmonary arterial pressure and mixed venous oxygen saturation [24]. To date, there are no available data comparing its efficacy and safety profile to standard MCS devices. Consequently, its use is not recommended in current consensus guidelines and is left to the availability at the center and the choice and expertise of the operators.

### 2.2. Proposed Decision Algorithm

To date, in the literature, there are no codified and validated decisional algorithms identifying CHIPs that are at higher risk of hemodynamic instability or peri-procedural complications and could most benefit from the employment of MCS.

According to Truesdel et al. [3], the choice between medical therapy, surgery, or PCI integrates clinical and anatomical factors inherent in the definition of high-risk PCIs as well as operators’ experience and guideline recommendations. Among these, the patient’s profile plays a pivotal role, with an increased burden of comorbidities being the main predictor of readmission within 30 days after an assisted PCI [25]. This is especially true in the management of elderly and frail patients, where the presence of cognitive decline or physical debilitation outweighs procedural complexity. In such cases, conservative medical treatment is indicated due to the risks associated with both PCI without MCS and potential complications associated with MCS itself.

Conversely, for other patients (e.g., non-frail elderly), the authors mention LV systolic dysfunction, decompensated heart failure, and other adverse hemodynamic conditions reducing physiologic reserve as key determinants influencing the choice of elective MCS. In this regard, Geppert et al. have recently proposed two comprehensive decision-making algorithms (with or without standby MCS for borderline situations) based on scores calculated from empirical patient and procedural variables that are awaiting validation in larger cohorts [26]. They assume that patients with severe systolic dysfunction or without a preserved cardiac index (2 points each) are at higher risk. These conditions may be accompanied by hemodynamic instability, acute coronary syndrome at presentation, electrical instability, pulmonary edema, or acute heart decompensation, as well as comorbidities including mitral regurgitation or severe aortic stenosis, chronic obstructive pulmonary disease, cerebrovascular disease, right ventricular failure, and procedural complexities. A score greater than 6 would result in the use of MCS being recommended, while a score between 4 and 5 would leave the decision up to the operator.

Of note, at the same time as considering the need for any type of MCS to be implanted, an assessment should be also made on the need to support hemodynamics with inotropic drugs or vasopressors. For example, initial intensive pharmacotherapy with an inodilator like Levosimendan combined with Impella CP support has been shown as a valuable strategy in severely compromised patients [27]. Finally, it is crucial to establish adequate vascular access, including central venous access for fluid or drug administration, and arterial access for continuous blood pressure monitoring as well as periodic evaluation of lactate levels and electrolytes.

## 3. Clinical Scenarios for the Use of MCS during CHIPs

The principal indications for MCS during CHIPs, as outlined in the latest clinical expert consensus document, are summarized in Table 2.

Overall, we can distinguish bail-out strategies if MCS is urgently employed (e.g., hemodynamic instability or slow-no reflow not promptly responsive to drugs) or prophylactic strategies when MCS is considered to avoid hemodynamic compromise in stable patients. In this section, we will discuss some of the most common scenarios in which MCS is employed.

### 3.1. Severe LV Dysfunction

Severe LV dysfunction poses significant challenges during complex PCI predisposing to worsening heart failure, hemodynamic compromise, and incomplete revascularization [28].

The rationale for using MCS in this scenario is based on several mechanisms.

First, transient ischemia occurring during PCI may further impair cardiac output in patients with underlying LV dysfunction. Thus, by maintaining adequate hemodynamics during the vulnerable period of the procedure, MCS may mitigate any procedure-related ischemic injuries [29].

Second, MCS may have also an impact on myocardial function recovery after a CHIP. The RESTORE EF study, involving 406 patients with a median baseline LVEF of approximately 35% and undergoing CHIP with Impella support, showed a significant improvement in LVEF at 90 days and a substantial reduction in symptom severity. Notably, the degree of LVEF improvement correlated with the extent of complete revascularization. However, this analysis included an ideal patient population with a 90-day 100% survival rate, thus limiting the generalizability of results [30]. The same findings were later confirmed by Romagnoli et al. in a very high-surgical-risk patient population. They found a significant association between extensive revascularization and global and segmental contractile improvement. Additionally, regional wall motion recovery was limited to revascularized segments and correlated with the severity of pre-procedural LV dysfunction [31].

### 3.2. Unprotected Left Main and Severe Multivessel Disease

Patients with unprotected left main (LM) disease and/or multivessel disease commonly have a poor prognosis, which is linked to the large amount of myocardium at risk and the often concomitant LV dysfunction [32,33].

In this context, the multidisciplinary heart team plays an even more critical role in the decision-making process due to the controversial results from recent long-term follow-ups of available trials comparing the effectiveness of CABG vs. PCI [34,35,36,37]. To determine the optimal revascularization strategy, the collegial discussion should consider the patient’s clinical characteristics, preferences, and coronary anatomy. This comprehensive approach ensures the best possible outcome. Additionally, when PCI is indicated, the best procedural approach and the need for, and type of, MCS should be part of the shared decision-making process.

Several studies have previously demonstrated the benefits and safety of MCS in an LM CHIP. Nearly 20 years ago, Briguori et al. pioneered this research by showing that IABP use can prevent intraprocedural events in high-risk patients undergoing LM stenting [38]. Similarly, the USpella registry reported low in-hospital (1.43%) and 30-day mortality rates (2.1%) in patients undergoing Impella-assisted LM PCI along with a significant percentage of complete revascularization (with average baseline and post-PCI SYNTAX scores of 31.4 and 7.86, respectively) [39]. Additionally, better short-term outcomes were observed when selecting Impella over an IABP in a small population of selected high-risk patients [40].

### 3.3. Chronic Total Occlusions

Chronic Total Occlusions (CTO) are at increased risk of complications due to the large amount of contrast needed, the prolonged procedural time, and the use of multiple equipment. Therefore, MCS devices may mitigate the risk of myocardial ischemia or injury by providing hemodynamic stabilization and limiting the episodes of hypotension and prolonged ischemia despite not being free from complications.

Indeed, in a retrospective analysis from five CTO referral centers, 57 of 2889 CTO PCIs (2%) received elective MCS with Impella 2.5 or CP. Impella-assisted CTO PCI was associated with high technical (87.7%) and procedural success (75.4%) rates along with a significant risk of peri-procedural complications, including vascular injury (5.3%), all-cause death (5.3%), major bleeding (3.5%), stroke (1.8%), and coronary perforation (1.8%) [41]. A similar technical and procedural success was reported in a single-center study involving 13 non-urgent TandemHeart-supported CTO PCIs (92% and 77%, respectively). Again, several complications, including vascular injury, coronary perforation, and one case of cardiogenic shock secondary to right ventricular wall hematoma, were observed [42].

The predictors of worse outcomes were age, previous congestive heart failure, moderate/severe calcification, use of retrograde crossing strategy, female gender, and lack of diabetes mellitus, as observed in a large multicenter registry of 7171 CTO PCIs (4.5% rate of MCS devices) [43].

### 3.4. Severely Calcified Disease Requiring Calcium Modification Techniques

Plaque modification and debulking strategies are essential when dealing with severe coronary artery calcification to prevent stent under-expansion and restenosis, which can lead to recurrent ischemic events.

Such strategies include balloon-based techniques such as non-compliant balloons, scoring balloons, cutting balloons, and intravascular lithotripsy (IVL), as well as calcium-ablation techniques like rotational atherectomy, orbital atherectomy, and laser atherectomy [44,45,46,47]. Sometimes a combination of techniques is needed, as anecdotally shown for orbital atherectomy and IVL or rotational atherectomy and IVL [48,49]. In such cases, distal embolization, slow flow or no-reflow phenomenon, vessel dissection, or perforations may occur, leading to hemodynamic crashes.

To date, there is no evidence of an absolute clinical benefit of MCS in this specific setting. Its use should be carefully discussed on a case-by-case approach, considering the baseline LV function, the type of calcium modification technique needed, and the risk of severe complications related to the MCS chosen.

### 3.5. Severe Concomitant Heart Valve Disease

The use of mechanical circulatory support (MCS) in patients with severe heart valve disease and concomitant coronary artery disease is largely unexplored.

In a recent US National registry that included approximately 60,000 patients undergoing transcatheter aortic valve replacement, only 2.8% received MCS in either emergent or elective settings. The IABP was the most commonly used (87%), followed by Impella and ECMO (6% each). Emergent MCS was associated with worse outcomes compared to elective use, presenting the conclusion that early identification of patients with aortic stenosis deemed at risk of hemodynamic compromise might rationalize elective MCS adoption [50].

This conclusion may also apply to Impella, although aortic stenosis is usually considered a relative contraindication. Further research is warranted, but a small registry of 21 patients has demonstrated its feasibility and safety in acting as support for PCIs after balloon aortic valvuloplasty in selected high-risk patients with severe aortic stenosis, with a 30-day mortality rate of 14.1% [51].

Once again, Almajed et al. suggested the importance of a heart-team-based approach, showing as examples a first case of LM calcific bifurcation treated with PCI under Impella support immediately after balloon aortic valvuloplasty and another case using peripheral Veno-Arterial ECMO to support distal LM PCI and subsequent transcatheter aortic valve replacement in a patient with concomitant severe LV dysfunction [52].

## 4. Risk and Complications of MCS Devices

While MCS devices provide beneficial hemodynamic effects, they carry a high risk of severe and life-threatening complications [9,53]. The causes are multifactorial and may be related to the large-bore access used for device insertion, the need for anticoagulation, any device malfunctioning, or detrimental homeostatic effects. Proper management requires not only a high level of technical skills but, once again, a collaborative and multidisciplinary organization.

### 4.1. Vascular Complications

Vascular complications are common in all MCS devices and correlate with higher in-hospital mortality and hospitalization costs [54]. Their incidence varies, ranging from 4% for IABP and 6–8% for Impella to 12–15% for ECMO, with a slight reduction being observed in the last decade due to increased operator expertise and improved practices for large-bore access management and closure [55].

Vascular issues typically involve limb ischemia or trauma during device insertion (such as dissection or laceration), which can result in a false aneurysm, hematomas, or hemorrhage. Predictors are large cannula size, female gender, systolic dysfunction, peripheral heart disease, and the need for vasopressors. All these factors must be considered before the procedure and included in the decision-making and assessment of the relative risks of PCI with or without MCS compared to CABG or medical therapy alone [56].

Once the indication for MCS is placed, best practices for large-bore access management should be followed. These include pre-procedural multi-modality imaging assessment to understand the anatomy of the common femoral artery, detect the extent and location of calcification, and plan any necessary pre-treatment for the ilio-femoral axis. The use of ultrasound-guided femoral puncture with the standard application of both transversal and longitudinal views is crucial [57]. This approach helps avoid a lower puncture height or puncture in calcified plaques and has been associated with a significantly higher first-pass success rate, albeit with contrasting data on its real effect on the reduction in vascular complications [58,59]. Alternative strategies are sometimes required, such as the SHiP (Single access for High-risk PCI) technique. This involves using a micro-puncture needle to perforate the diaphragm of the Impella peel-away sheath and place a 7 Fr sheath through the hemostatic valve and adjacent to the 9 Fr Impella catheter shaft, through which complex PCI can be performed [60].

Regarding vascular closure methods, there is no universally accepted best practice. The most commonly used approach is “pre-closure”, which involves pre-implanting one or two suture-mediated devices, typically positioned at the 10 and 2 o’clock configurations [61]. Alternatively, if a post-closure strategy is preferred, several options are available. These include a double-wire technique with two suture-mediated devices, a “hybrid” method combining one suture-mediated device and a vascular plug, or even balloon hemostasis from contralateral access [62].

In addition to access site-related issues, MCS devices can compromise abdominal visceral or cerebrovascular perfusion. This is especially common with intra-aortic balloon pumps (IABPs) and is often due to incorrect sizing or improper positioning. Finally, although rare, dislodgement of a proximal thrombus from any device surface may cause embolization, which is more likely to result in visceral or limb ischemia, infarction, or stroke [63].

### 4.2. Contrast Associated Acute Kidney Injury

Contrast-associated acute kidney injury (CA-AKI) is a common complication following PCI, with an elevated risk in patients with pre-existing renal impairment, and has been associated with irreversible deterioration of renal function, the need for in-hospital renal replacement therapy or dialysis, and death [64]. Although its incidence in patients undergoing CHIPs has not been extensively studied, it is expected to be significant considering the higher direct toxicity due to the amount of contrast medium required and the indirect mechanism of vasoconstriction causing medullary hypoperfusion and ischemic injury.

Interestingly, a retrospective study by Azzalini et al., despite failing to show any difference in the rate of CA-AKI among complex and non-complex PCI patients [65], recognized certain clinical features, such as acute coronary syndrome at presentation and specific comorbidities, as predictors for developing renal impairment following PCI [66].

Therefore, stratifying the risk of CA-AKI using a practical and well-standardized algorithm which combines readily available clinical factors and procedural variables, such as the Mehran score, may help identify patients who would benefit most from preventive strategies or possibly preventive continuous renal replacement therapy [67]. However, strategies to prevent renal damage should be adopted for all patients. These include limiting the maximum amount of total contrast volume (in mL) to estimated glomerular filtration rate (in mL/min) [volume to creatinine clearance should be less than 3.7] [68], using low-osmolar or iso-osmolar contrast media, and providing pre-and post-hydration with isotonic saline solution [69]. Additional strategies, such as the administration of acetylcysteine, do not have sufficient evidence for recommendation.

### 4.3. Hemolysis

The higher risk of hemolysis is observed in 5–10% of patients treated with Impella [11]. This complication can be pump-related, patient-related, or a combination of both [53].

Increased shear stress on the device’s surface is an important determinant of erythrocyte damage. This stress depends on the number of rotations per minute, which is much higher with Impella 2.5 compared to Impella CP (51,000 rotations per min vs. 33,000 rotations per min, respectively). Other determinants include inadequate anticoagulation of the purge flow solution, improper positioning of the device, and any causes of suction determining inflow obstruction, such as reduced pre-load, hypovolemia, right ventricular dysfunction, or vasoplegia [70,71]. The impact of hemolysis on mortality is unclear, but it is certainly associated with elevated plasma levels of free hemoglobin and hyperbilirubinemia, ultimately leading to acute kidney injury [72,73,74].

Once hemolysis is clinically suspected, it is mandatory to check device positioning and, if dislodged, reposition it under fluoroscopic and echocardiographic guidance [75].

When mispositioning is ruled out, it is essential to assess right ventricular function and any potential causes of hypovolemia. If right ventricular dysfunction is identified, inotropes or right ventricular MCS may be beneficial. In cases of hypovolemia, a fluid challenge is crucial for empirical diagnosis and treatment [76,77]. When any anticoagulation issues are excluded, other systemic causes of hemolysis should be considered and addressed [78].

### 4.4. Anticoagulation Management: Balancing between Thromboembolic and Bleeding Events

Thromboembolic and bleeding complications are common causes of morbidity and mortality, especially when MCS weaning does not occur immediately after the procedure [79].

Anticoagulation, typically with unfractionated heparin, is imperative to minimize clotting of the circuit and reduce the risk of device-related thromboembolic events [80]; however, when it is combined with the usual need for antiplatelet therapy after PCI, it results in a significant bleeding risk [81,82]. This may be further exacerbated by the acquired von Willebrand syndrome, which derives from the proteolysis of high molecular weight von Willebrand factor due to shear stress and continuous flow within the MCS, thus reducing platelet binding affinity and enhancing the tendency to bleed. The syndrome usually develops after 24 h of MCS use, and the only solution is device removal [83]. Major bleeding is reported in up to 80% of patients on Veno-Arterial ECMO, with approximately 16% experiencing intracranial hemorrhage [84]. Moreover, it is higher in patients undergoing Impella support compared to those using IABP, as demonstrated in two retrospective propensity-matched studies of patients with cardiogenic shock or CHIPs in the United States [85,86].

The meticulous monitoring of the unfractionated heparin regimen is essential. Activated clot time is widely available, but its use outside the catheterization laboratory is not recommended due to its high variability. Activated-partial-thromboplastin-time and, if available, anti-Xa assays are usually preferred in patients needing prolonged mechanical support after the procedure.

## 5. Unmet Needs and Future Directions

The continuous evolution of interventional cardiology, driven by the development of sophisticated techniques and materials with ever-increasing handling profiles, has made previously untreatable anatomies addressable. However, the following paradox persists: the highest-risk patients, who would benefit most from PCI, always remain the less likely to receive revascularization. This happens because, while the patients’ complexity is increasing, technical progress is not equally distributed across all the centers.

This highlights the need to establish standards of expertise, develop algorithms for managing the most complex cases in high-volume, specialized centers, and promote training programs in smaller low-volume hospitals.

In this regard, Pietrasik et al. have proposed a 10-step model for the implementation of an institutional Impella program [87]. It suggests following a standard operating procedure of patient selection based on heart team decisions, pre-procedural assessment of peripheral artery disease severity, potential LV thrombi (or any other contraindication for MCS), and a procedural plan that involves choosing optimal large-bore access, discussing PCI strategies and alternatives, and selecting the most experienced operator. Starting with high-risk PCI support and preferring femoral access in patients without extensive PAD is recommended, as is multidisciplinary post-procedural care using continuous, careful hemodynamic and laboratory monitoring to promptly diagnose access-site complications, bleeding, hemolysis, acute kidney injury, and infections. This algorithm also includes dedicated training sessions for inexperienced operators, embodying the concept of “CHIP fellowships”.

These structured one-year training programs offer extensive exposure to complex procedures (such as bifurcation, CTO, and LM PCI) as well as mentored research opportunities, both crucial in a field where expertise is lacking and many of the indications and techniques are currently evolving. The primary goal is to prepare fellows to independently perform complex procedures while minimizing the rate of complications and promoting a fair relationship with mentors, which is key to long-term success [88].

Beyond operator expertise, advancements in technology are crucial for improving the management of CHIPs. Recent examples are the new Supira system, with its better profile, multiple sensors for real-time monitoring of pump performance, and smaller sheaths to minimize vascular complications, as well as new devices equipped with advanced artificial intelligence software. Once the challenges related to developing reliable models that can continuously adapt their predictive power to the dynamic evolution of MCS patients’ clinical states are overcome, these AI tools may represent a breakthrough that has an extraordinary impact on the clinical world of CHIP PCIs.

## 6. Conclusions

CHIPs are associated with higher morbidity, mortality, and procedural failure due to the risk of patients’ hemodynamic destabilization. Percutaneous MCS devices have been developed to assist with PCIs in these critical scenarios but can be harmful if adopted inappropriately. A multidisciplinary heart team is essential in evaluating every single patient and determining who will most benefit from MCS. At the same time, further research is warranted to better understand the efficacy and safety profiles of these devices and identify those technical gaps where the expertise of the interventional cardiologist needs improvement and additional technical advancements are still required.

## Figures and Tables

**Table 1:** Advantages and disadvantages of different Mechanical Circulatory Support Devices.

Device	Advantages	Disadvantages	Indications
IABP	Easy insertion and low risk of complications.	Slight hemodynamic support inadequate in case of severe or inotropic-refractory cardiogenic shock.	○ Patients with mild-moderate hemodynamic instability; ○ LVEF > 30%; ○ Short or less complex coronary procedure; ○ Contraindications to more supportive MCS.
Impella	Greater hemodynamic support and possibility of choosing the appropriate flow rate.	Complex insertion procedure high risk of complications.	○ Patients with severe cardiogenic shock; ○ LVEF < 30%; ○ Complex and prolonged coronary procedure.
ECMO	Maximum support for patients with severe cardiac and respiratory failure.	Complex insertion procedure high risk of complications.	○ Patients with refractory cardiogenic shock and concomitant respiratory failure; ○ Need for short-term maximum support.
TandemHeart	Robust hemodynamic support.	Complex insertion and procedure technical experience required to perform transeptal puncture and high risk of complications.	○ Patients with cardiogenic shock refractory to inotropes; ○ Need for prolonged circulatory support; ○ LVEF < 20%.
PulseCath iVAC	Increase the mean arterial pressure while not influencing mean pulmonary pressure and mixed venous O_2_ saturation.	Complex insertion procedure high risk of complications.	○ Patients with severe cardiogenic shock; ○ Left ventricular support for up to 24 h; ○ Complex and prolonged coronary procedure.

Abbreviations: LVEF, left ventricular ejection fraction; MCS, mechanic circulatory support; IABP, intra-aortic balloon pump; ECMO, extracorporeal membrane oxygenation.

**Table 2:** Principal indication of MCS according to the clinical scenario, summarized from last 2019 clinical expert consensus [4].

Scenario	Recommendation
Unprotected Left Main and Severe Coronary Artery Disease	Percutaneous MCS is strongly encouraged in case of unprotected distal LM associated with SYNTAX score ≥ 33 and severe LV dysfunction (LVEF ≤ 35%) when surgical approach is not possible. Consider a Percutaneous MCS in case of non-emergent CHIP for unprotected distal LM associated with SYNTAX score > 22 and severe LV dysfunction (LVEF < 35%).
Complete Revascularization	Percutaneous MCS pre PCI is indicated in patients undergoing non-emergent CHIP, in case of complex procedures in patients with severe LV dysfunction in the attempt to obtain complete revascularization.
Complex CTO	Percutaneous MCS is indicated as preventive strategy in symptomatic or ischemic patients with (1) severely reduced LVEF and complex anatomical setting, if not amenable for surgery, including high-risk CTO features; (2) less than severe LVEF and complex anatomical settings as second attempt after a failed CTO-PCI because of hemodynamic instability or CTO-PCI retrograde from last remaining vessel.
Last Remaining Vessel	Percutaneous MCS protected PCI is strongly indicated as life-saving strategy, in last remaining vessel revascularization non-amenable for CABG associated with LVEF dysfunction.
Diffuse and Calcified Lesions	Percutaneous MCS non-emergent protected PCI is indicated in patients at risk because of the coronary disease and severe LV dysfunction especially when rotational atherectomy is required.
High-Risk Slow-No Reflow	Percutaneous MCS use in this setting is indicated as bail-out strategy in case of slow-no reflow not promptly responsive to drugs and associated with hemodynamic decay of the CHIP patients.
Hemodynamic Instability	Apply Percutaneous MCS in hemodynamic instability to prevent or support hemodynamic compromise.
Severe LV Dysfunction and Heart Failure	Percutaneous MCS should be considered in a heart team approach and should be implanted prior to intervention in an effort to avoid “crashing onto support” and to enable complete revascularization when feasible in patients without a surgical revascularization option.
Severe Concomitant Heart Valve Disease	Percutaneous MCS use in patients with severe heart valve disease is actually not recommended. Percutaneous MCS bail-out use to stabilize patients who crushed after aortic valvuloplasty may be considered in experienced centers when further valve treatments are considered feasible.

Abbreviations: LM, left main; LV, left ventricular; MCS, mechanical circulatory support; PCI, percutaneous coronary intervention; CTO, chronic total occlusion; CHIP, complex and high risk indicated PCI; LVEF, left ventricular ejection fraction; CABG, coronary artery bypass graft.

## Data Availability

Data sharing is not applicable to this article as no new data were created or analyzed in this study.
